# The CHARGE Study: An Epidemiologic Investigation of Genetic and Environmental Factors Contributing to Autism

**DOI:** 10.1289/ehp.8483

**Published:** 2006-04-06

**Authors:** Irva Hertz-Picciotto, Lisa A. Croen, Robin Hansen, Carrie R. Jones, Judy van de Water, Isaac N. Pessah

**Affiliations:** 1 Division of Epidemiology, Department of Public Health Sciences, School of Medicine and; 2 Medical Investigations of Neurodevelopmental Disorders (MIND) Institute, University of California–Davis, Davis, California, USA; 3 Division of Research, Kaiser Foundation Research Institute, Kaiser Permanente, Oakland, California, USA; 4 Department of Pediatrics and; 5 Department of Rheumatology/Allergy and Clinical Immunology, School of Medicine and; 6 Department of Molecular Biosciences, School of Veterinary Medicine, University of California–Davis, Davis, California, USA

**Keywords:** autism, autistic spectrum disorder, developmental delay, environment, genetics, mental retardation, pervasive developmental disorder

## Abstract

Causes and contributing factors for autism are poorly understood. Evidence suggests that prevalence is rising, but the extent to which diagnostic changes and improvements in ascertainment contribute to this increase is unclear. Both genetic and environmental factors are likely to contribute etiologically. Evidence from twin, family, and genetic studies supports a role for an inherited predisposition to the development of autism. Nonetheless, clinical, neuroanatomic, neurophysiologic, and epidemiologic studies suggest that gene penetrance and expression may be influenced, in some cases strongly, by the prenatal and early postnatal environmental milieu. Sporadic studies link autism to xenobiotic chemicals and/or viruses, but few methodologically rigorous investigations have been undertaken. In light of major gaps in understanding of autism, a large case–control investigation of underlying environmental and genetic causes for autism and triggers of regression has been launched. The CHARGE (Childhood Autism Risks from Genetics and Environment) study will address a wide spectrum of chemical and biologic exposures, susceptibility factors, and their interactions. Phenotypic variation among children with autism will be explored, as will similarities and differences with developmental delay. The CHARGE study infrastructure includes detailed developmental assessments, medical information, questionnaire data, and biologic specimens. The CHARGE study is linked to University of California–Davis Center for Children’s Environmental Health laboratories in immunology, xenobiotic measurement, cell signaling, genomics, and proteomics. The goals, study design, and data collection protocols are described, as well as preliminary demographic data on study participants and on diagnoses of those recruited through the California Department of Developmental Services Regional Center System.

Autism is a serious neurodevelopmental disorder characterized by impairments in social interaction, abnormalities in verbal and nonverbal communication, and restricted, stereotyped interests and behaviors (American Psychiatric Association 1994). Although a large proportion of individuals with autism manifest abnormal development from birth, a subset of at least 20–30% experience a regression with onset between 18 and 24 months of age after a period of apparently normal development (Lainhart et al. 2002). Autistic disorder is the most severe form of autism spectrum disorders (ASDs), which include Asperger’s syndrome and pervasive developmental disorders (PDDs) not otherwise specified. Approximately 70% of individuals with autistic disorder have some degree of mental retardation, and about half are nonverbal or have very impaired speech. Seizures are present by adolescence in about 30% of children with ASD, and between 5 and 10% of autism cases occur in association with other serious medical conditions such as fragile X, tuberous sclerosis, and Angelman’s syndrome (Fombonne 2003). Gastrointestinal problems and sleep disturbances are also thought to be common comorbidities; however, population-based prevalence estimates for these conditions are currently lacking. Males are four times as likely as females to have autism, but this ratio approaches one among individuals with severe cognitive impairment (Gillberg and Wing 1999). Most individuals with autism cannot live independently as adults (Rapin 1999). Over the past 20 years, the prevalence of autism has reportedly risen, with much public debate surrounding the reasons for this increase. Early reports estimated prevalence at 4–5 per 10,000 births (Fombonne 1999). Data published in the last few years suggest that autistic disorder occurs in at least 1–2 per 1,000 births, and the prevalence of the broader autism spectrum may be as high as 4–6 per 1,000 (Chakrabarti and Fombonne 2005; Yeargin-Allsopp et al. 2003).

The causes and contributing factors for autism are poorly understood. The number of children with a diagnosis of autism as determined by the California Department of Developmental Services (DDS) has been rising continuously for over a decade (California DDS 2003). Although diagnostic changes and improvements in detection probably contribute to this increase (Chakrabarti and Fombonne 2005; Croen et al. 2002), a true rise in incidence may also be occurring (Blaxill et al. 2003). Evidence for genetic causes is strong, yet concordance in monozygotic twins suggests that a minimum of 40% of autism cases are likely to have an environmental cause. No single gene has yet been specifically linked to autism with replicability, but the disorder is believed to be polygenic. A few specific environmental factors are associated with autistic behaviors—prenatal exposures to thalidomide (Rodier and Hyman 1998), valproic acid (Christianson et al. 1994), or rubella (Chess et al. 1978)—but these are likely to play a negligible role, if any, in incident cases in Western countries over the last decade or so.

Mechanisms of pathogenesis have yet to be delineated. Contrary to early beliefs that autism resulted from bad parent–child interactions (Bettelheim 1967), it is now widely accepted that aberrant brain development underlies autism pathogenesis (Bauman and Kemper 2003; Courchesne et al. 1988; Piven et al. 1990; Rodier et al. 1997). Autopsy studies demonstrate structural changes in the brain, and imaging and electrophysiology investigations reveal neurophysiologic differences in information processing between children with autism and those with typical development (Dawson et al. 2002; Maziade et al. 2000; McPartland et al. 2004; Rapin and Dunn 2003; Rosenhall et al. 2003). Neuroimmunomodulatory factors may also play a role (Silva et al. 2004; Vargas et al. 2005). Cytokine profiles, lymphocyte activation, and other immunologic parameters differ between individuals with and without autism (Ashwood and Van de Water 2004a, 2004b; Croonenberghs et al. 2002). Distributions of neuropeptides and neurotrophins at birth appeared to be altered among children who later developed autism (Nelson et al. 2001).

Results from twin and family studies suggest a strong genetic contribution to the etiology of autism. Beginning with the classic work by Folstein and Rutter (1977), data from three population-based twin studies have demonstrated a higher concordance rate among monozygotic compared with dizygotic twins (Cook 1998). Strong familial aggregation of autism has also been demonstrated. The sibling recurrence risk (i.e., the probability of developing autism given a person’s sibling is autistic) has been estimated at 2–14% (Jorde et al. 1990; Ritvo et al. 1989; Smalley et al. 1988), a 10- to 20-fold increase over the general population prevalence. A family history of social deficits, language abnormalities, and psychiatric disorders has also been observed in case–control and clinic-based studies (Folstein and Rutter 1988; Piven and Palmer 1999).

Autism co-occurs with several known genetic disorders, such as tuberous sclerosis (Smalley 1998), Angelman syndrome (Steffenburg et al. 1996), phenylketonuria, Joubert syndrome (Ozonoff et al. 1999), and Möbius syndrome (Johansson et al. 2001), and chromosomal abnormalities such as fragile X syndrome (Reiss and Freund 1990). More than 90% of autism cases, however, have none of the above syndromes.

Linkage, association, and cytogenetic studies have been conducted. Numerous candidate genes for autism have been suggested based on their functional role, location within candidate chromosome regions, and positive associations with the disease (Korvatska et al. 2002). Replication of findings has been elusive (Wassink et al. 2004), probably because of the polygenic etiology, heterogeneity of the phenotype, and, assuming a role for gene–environment interaction, variation in exposure distributions across populations. An epigenetic mechanism related to Rett syndrome is also plausible (Samaco et al. 2005). Genomewide scans to identify regions marked by differing gene expression are considered key at this stage. One such scan hints at the possible genetic basis for the well-established sex ratio of four males to one female (Stone et al. 2004). A comparison of tuberous sclerosis patients with and without autism demonstrated 31 genes for which expression differed (Tang et al. 2004); because both groups shared the tuberous sclerosis diagnosis, the differentially expressed genes may be related to autism, although they are not necessarily causal. It is plausible that a substantial proportion of autism cases could be due to multiple genes interacting with one or more environmental factors (Cederlund and Gillberg 2004; Glasson et al. 2004).

Neuroanatomic and epidemiologic investigations support a prenatal or early postnatal origin. Courchesne et al. (1988) observed cerebellar abnormalities consistent with abnormalities in cell migration between the third and fifth month of gestation. Magnetic resonance imaging studies point to migrational errors that result in disorganized columns of the cerebral cortex (Casanova et al. 2002). Anthropometric indicators, such as brain size and growth trajectory (Herbert 2005), suggest overall cerebral volume to be larger in mid-childhood, with growth that accelerates early and then decelerates, although this phenotype may apply to only a subset of cases. Neuroimaging studies indicate involvement of specific brain regions, including the amygdala, hippocampus, and corpus callosum (Brambilla et al. 2003; Schumann et al. 2004).

Studies of environmental factors also relate to the prenatal origin of autism. Chess et al. (1978) reported that, within a cohort of about 250 children with congenital rubella, 7% were later diagnosed with autism. A case–control study using both maternal reports and medical records of illnesses during pregnancy showed relative risks of 4.1 for influenza and 3.3 for rubella (Deykin and MacMahon 1979). Daily maternal smoking during early pregnancy was reported to be linked to autism in a large case–control epidemiology study (odds ratio = 1.4; 95% confidence interval, 1.1–1.8) (Hultman et al. 2002), although in our estimation, these analyses may have inappropriately adjusted for potentially intermediate variables. The link between autism and early *in utero* exposure to thalidomide places the timing of the insult coincident with neural tube closure in the fourth to fifth week of gestation (Rodier and Hyman 1998). Case reports of autism in children gestationally exposed to valproic acid (Christianson et al. 1994; Rodier et al. 1997; Williams et al. 2001) are concordant with experimental animal studies (Ingram et al. 2000). A small number of cases of autism after maternal infection with cytomegalovirus (Markowitz 1983; Stubbs et al. 1984), measles or mumps (Deykin and MacMahon 1979), or herpes (Ritvo et al. 1990) as well as one case each of syphilis and toxoplasmosis (Rutter and Bartak 1971) have been reported.

Taken together, the literature suggests a prominent genetic component involving multiple gene loci, but also a likely contribution from both chemical and microbial agents. It is likely that further understanding will require consideration of critical windows during gestation and possibly early infancy, as well as interactions between genetic or epigenetic predisposition and environmental factors.

## CHARGE Study Aims

In light of the enormous gap in our understanding of the causes of both autism and developmental delay (DD), a large epidemiologic study was initiated in 2002. The Childhood Autism Risk from Genetics and the Environment (CHARGE) study is addressing a wide spectrum of environmental exposures, endogenous susceptibility factors, and the interplay between these two (CHARGE 2006).

**Figure 1:**
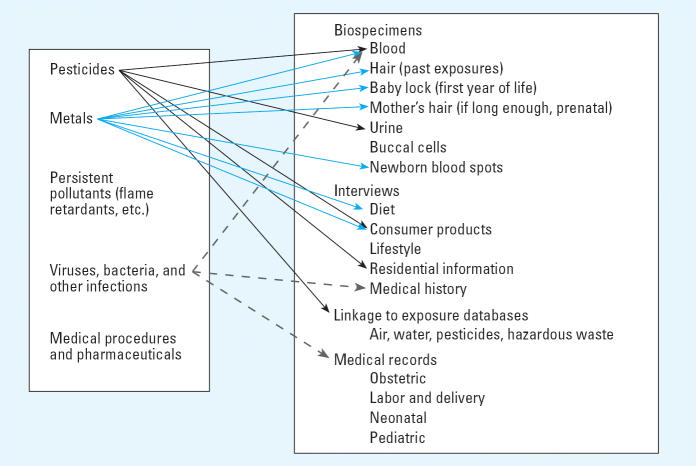
Environmental exposures and sources of information in the CHARGE study. The left-hand box indicates five classes of exposures that are candidates as environmental factors contributing to autism. The right-hand box lists sources of data available on CHARGE study participants. Arrows show a few examples of how specific exposures can be assessed. For example, pesticide exposures and/or their metabolites can be assessed in several ways (black arrows): laboratory assays can be conducted on blood (serum) and urine specimens; the interview collects information on applications in the home and also obtains residential histories that can be linked to exposure databases on commercial pesticide applications in California. Metals (blue arrows) can be measured in hair and in newborn blood spots obtained from the State Genetics Diseases Branch biospecimen bank or assessed by interview questions on fish consumption or use of household products. Exposures to infectious agents (dashed arrows) can be determined from medical records, self-reports, and assays on serum samples to test for seropositivity for antibodies to specific viruses.

To structure the search for etiologic factors, we are beginning with known neurodevelopmental toxicants and hints from the immunologic evidence. Additionally, physiologic differences that might provide clues about susceptibility and mechanisms are being examined through characterization of metabolic, immunologic, and gene expression profiles, as well as genetic polymorphisms. Figure 1 shows five broad classes of exposures of interest: pesticides, metals, persistent pollutants with known or suspected neurodevelopmental or immunologic toxicity, medications and other treatments, and infections. Exposures from both the prenatal and early childhood periods are being investigated, with data primarily from three sources: *a*) extensive interviews with parents; *b*) laboratory analysis of xenobiotics in blood, urine, and hair specimens; and *c*) prenatal, labor and delivery, neonatal, and pediatric medical records.

CHARGE study specimens are analyzed for immunologic, cell activation, xenobiotic, lipomic, and genomic markers in laboratories of the University of California–Davis (UC Davis) Center for Children’s Environmental Health (CCEH) (Table 1). Metals have been assayed in blood samples from > 300 index children, with a focus on mercury, lead, arsenic, cadmium, and manganese. Immunologic profiles are being characterized, including cellular responses to bacterial antigenic stimulation, general immunoglobulins, and production of chemokines and cytokines. Already, preliminary results have demonstrated significant differences between children with autism and children from the general population in leptin concentrations (Ashwood P, Kwong C, Hansen R, Hertz-Picciotto I, Croen L, Krakowiak P, et al., unpublished observations).

A detailed lipomics screen is being applied to the plasma from the first few hundred children. Affymetrix GeneChip microarrays (Affymetrix, Santa Clara, CA) have been generated from an initial sample of children and analyzed to determine whether a genomic fingerprint for autism can be identified; results will be replicated on a further set. Brominated flame retardants are being measured in 80–100 children, and metabolites of pyrethroid pesticides will be evaluated in urine specimens.

The CHARGE study also benefits from CCEH hypothesis-driven experimental research on animal models for autism in mice and non-human primates and *in vitro* investigations of immune and neurogenic cells aimed at uncovering molecular mechanisms. A common database coordinates the archival, retrieval, and analysis of samples, and the combination of population-based epidemiology with state-of-the-art molecular and cellular methods provides a powerful basis for interdisciplinary collaborative research. With future funding, the CHARGE study will undertake targeted evaluation of candidate genes, such as those responsible for regulation of xenobiotic metabolizing enzymes, cell signaling in both neurons and immune cells, and immune cell activation.

Currently, the study is also characterizing phenotypic variation within the autism case group and relating these phenotypes to the exposures and physiologic profiles of interest. For example, we have begun to compare immune function in regressive autism (children who have lost previously acquired social or language skills) with those with early onset (children who never acquired those skills). Other phenotypic subtypes include, for example, high versus low cognitive function and presence versus absence of gastrointestinal symptoms, macrocephaly, and sleep disturbances.

## Design and Subject Recruitment

The CHARGE study appears to be the first large-scale, population-based epidemiologic investigation focusing primarily on environmental exposures, as well as their interactions with genes, as underlying causes for autism. It uses the case–control design, which provides the most efficient sampling for studies of conditions that are rare or of multifactorial etiology. A further advantage is the focus on a specific outcome, which translates into close scrutiny of diagnoses and rigorous measurement for the most highly suspect risk factors.

The CHARGE study population is sampled from three strata: children with autism (full-syndrome autism, not those with a “spectrum disorder”), children with DD but not autism, and children selected from the general population without regard for developmental characteristics. All participating children (currently > 500, with an ultimate goal of between 1,000 and 2,000) meet the following criteria: *a*) between the ages of 24 and 60 months, *b*) living with at least one biologic parent, *c*) having a parent who speaks English or Spanish, *d*) born in California, and *e*) residing in the catchment areas of a specified list of regional centers (RCs) in California. No further exclusions are made based on genetics or family phenotype.

Children with autism and children with mental retardation or DD are identified through RCs that contract with the California DDS to determine eligibility and coordinate services for persons with developmental disabilities. Eligibility in the DDS/RC system does not depend on citizenship or financial status. Thus, the system is widely used across socioeconomic levels and racial/ethnic groups. Referrals are from pediatricians, other clinical providers, schools, friends, and family members.

The DDS/RC system is mandated to provide services for individuals with autism, as well as for those with other PDDs who have mental retardation (IQ < 70) or are substantially handicapped. One investigation estimated that 75–80% of the total population of children with an autism diagnosis in the state were enrolled in the DDS system (Croen et al. 2002). Among preschoolers, the figure may be lower, with fewer mild cases. Additionally, this proportion may decline with recent changes to eligibility requirements that emphasize the extent of disability. Children with Asperger’s or PDDs not otherwise specified without mental retardation are not generally eligible for DDS/RC services and therefore are not actively recruited into the CHARGE study.

Potential cases of autism for the CHARGE study are defined as those who are eligible for services based on a DDS/RC diagnosis of autism. Families with a child who has received a diagnosis but is not in the RC system are also invited. The second study group, children with DD, is likewise drawn from those determined eligible for services based on a diagnosis of mental retardation or DD. Children 0–3 years of age who are at risk for DD or disability can receive RC services under the Early Start program and are also eligible to be in the second CHARGE study group. The DD children must meet the above inclusion criteria but are not age- or sex-matched to the children with autism.

Staff of the RCs contact parents of children with autism or DD, provide them an information packet, and explain how they can participate in the CHARGE study. For those who are interested, permission is obtained for the study staff to telephone the families and schedule appointments. The children then undergo further testing (see below) to confirm their diagnoses.

The third group consists of children from the general population identified from state birth files. Throughout the study, we generate random samples of children meeting the study eligibility criteria according to their birth certificate information. This group is frequency-matched to the age, sex, and broad residential RC catchment area distribution of the autism cases. Using names and social security numbers in birth certificate files, study personnel attempt to locate current contact information and then initiate a recruitment effort.

### Data collection protocols

Participation involves assessments of cognitive and social development, a medical examination, biologic specimen collection, and completion of an exposure interview and several self-administered questionnaires. Other components include maternal and child medical records review and abstractions. Table 2 summarizes the protocols, other than specimen and medical record collection.

CHARGE study children are assessed at the UC Davis Medical Investigations of Neurodevelopmental Disorders (MIND) Institute; a small percentage were seen at the UCLA Neuropsychiatric Institute. Standardized clinical assessments are administered to confirm the child’s diagnostic group. Autism cases are assessed using diagnostic tools widely accepted for research: the Autism Diagnostic Interview–Revised (ADI-R) (Le Couteur et al. 2003; Lord et al. 1994, 1997) and the Autism Diagnostic Observation Schedules (ADOS) (Lord et al. 2000, 2003). The ADI-R is a standardized, semistructured 2- to 3-hr interview with caregivers of individuals with autism or PDDs. It yields summary scores in the following domains: qualitative impairments in reciprocal social interaction, communication, and repetitive behaviors and stereotyped patterns. Published values for interrater reliability are good, with kappa values ranging between 0.62 and 0.89 (Lord et al. 2003).

The ADOS is a semistructured, standardized assessment of children in which the examiner observes the social interaction, communication, play, and imaginative use of materials. The ADOS requires approximately 30 min and includes four possible modules; the examiner chooses the one that best matches the expressive language level of the individual child to prevent a relatively low level of language ability from impeding accurate measurement. Diagnostic algorithms are available for autism or for broader ASDs/PDDs (Lord et al. 2003). The ADOS provides measures in the following domains: reciprocal social interactions, communication, stereotyped behaviors and restricted interests, and play. All kappa values for interrater reliability exceeded 0.60. All CHARGE clinical assessment personnel are trained and have attained research reliability on the ADI-R and the ADOS.

Cognitive function is measured in all children (those with autism or DD and the general population controls) using the Mullen Scales of Early Learning (MSEL) (Mullen 1995). The MSEL is a standardized developmental test of children 3–60 months of age. The MSEL consists of five subscales: gross motor, fine motor, visual reception, expressive language, and receptive language. The MSEL allows for separate standard verbal and nonverbal summary scores to be constructed. The five MSEL scales demonstrate satisfactory internal consistency (0.75–0.83), internal reliability (0.91), test–retest reliability (0.71–0.96), and inter-rater reliability (0.91–0.99) (Mullen 1995).

Adaptive function is assessed by parental interview using the Vineland Adaptive Behavior Scales (VABS) (Sparrow et al. 1984). The VABS is the most widely used instrument for assessment of adaptive behavior across the lifespan and covers the domains of socialization (interpersonal relationships, play and leisure time, and coping skills), daily living skills (personal, domestic, and community skills), motor skills (gross and fine motor), and communication (receptive, expressive, and written communication), with developmentally ordered skills for each area. The scale is norm referenced, and recent supplemental norms have been published for individuals with autism (Carter et al. 1998). Psychometric properties of the instrument include excellent internal consistency (0.90–0.98), test–retest reliability (*r* = 0.78–0.92), and interrater reliability (*r* = 0.87 for young children).

Before the clinic visit, the parent is mailed the consent form to review and several self-administered forms to complete, including the Aberrant Behavior Checklist, a standardized checklist constructed to rate inappropriate and maladaptive behaviors in developmentally delayed individuals (Aman and Singh 1994); Multiple Language Questionnaire to determine what languages are used at home; Child Development Questionnaire (CDQ), consisting of 31 questions regarding acquisition and loss of language and skills, a subset of the Early Development Questionnaire (Ozonoff et al. 2005) to examine loss of developmental skills; and structured questionnaires about gastrointestinal symptoms and sleep habits of the child (developed *de novo*). Parents are also sent a list of autoimmune diseases with a description of each, so that they can prepare to respond to questions about family history of these disorders during the clinic visit. All instruments and forms are administered in either English or Spanish, depending on the language in which the parent or child feels most comfortable. The CHARGE study employs trained bilingual/bicultural staff for every phase of the study.

At the clinic, the psychometric assessments are administered, a family medical history with an emphasis on mental health and autoimmune disorders is taken, and a family characteristics questionnaire is used to document developmental and other aspects of the broader phenotype in immediate family members. Physical and neurologic exams are completed; dysmorphology and growth or neurologic abnormalities are recorded. Finally, blood specimens are collected at the end of the clinic visit. The parent is asked to bring in urine specimens for the child and immediate family members.

For families of children recruited from the nonautistic groups, the protocol is essentially identical, except that the ADI-R and ADOS are not routinely administered. The Social Communication Questionnaire (SCQ) was developed from the ADI-R to screen children for evidence of features of ASDs. If the score on the SCQ is above 15, the ADI-R and ADOS are administered on a second visit.

Final autism case status is defined as meeting criteria on the communication, social, and repetitive behavior domains of the ADI-R and scoring at or above the total cutoff for autistic disorder on the ADOS module 1 or 2. Analyses will be conducted for cases meeting criteria for autistic disorder, as well as for a broader definition of impairment encompassing ASDs. A similar approach will be used for mental retardation/DD: Children obtaining an MSEL composite score of < 69 and a VABS composite score of < 70 will be classified as meeting strict criteria for DD.

Separate from the clinic visit, we conduct a telephone interview with the primary caregiver regarding periconceptional, prenatal, and early childhood exposures and experiences. The interview of approximately 1 hr 40 min covers the following areas: demographics; mother’s medical history; mother’s reproductive and pregnancy history; index pregnancy, including use of reproductive technology for conception; maternal illnesses and medications during index pregnancy; metals, diet, and household product use; child’s illnesses and medications; maternal lifestyle information; residential history; and occupational history of the mother and father. An index time period is defined as 3 months before pregnancy to the end of pregnancy or, if the child was breast-fed, until weaning. Information on medications, metals, household products, and the occupational and residential histories focuses on this index period.

Blood and urine specimens are collected from the index child, parents, and siblings. For any family member from whom blood is not obtained, an attempt is made to collect buccal swabs for DNA extraction. Hair specimens are collected from the index child and from the mother if her hair is long enough to potentially contain information about exposures during the pregnancy or lactation period. If the parent saved locks from the child’s first haircut, we request a few strands. Additionally, neonatal blood spots from the index child will be obtained from the newborn screening specimen archive maintained by the Genetic Disease Branch of the California Department of Health Services (Richmond, CA).

Medical records are procured and abstracted for information about procedures, medications and other treatments, and conditions at birth of the index child. Obstetric/gynecology/prenatal clinic and mental health provider records are obtained for the mother. Similarly, labor and delivery, neonatal, pediatric, and specialty clinic medical records are procured. Dental records are sought for the confirmation of mercury amalgams.

The study complies with all applicable requirements regarding human subjects and is approved by the institutional review boards for the State of California and the University of California. Informed consent is obtained before collection of any data.

### Preliminary data on participants and their diagnoses

Full recruitment into the CHARGE study began in late 2003. More than 520 children and their families have enrolled in the CHARGE study at the time of this writing. This includes > 360 recruited because of an RC diagnosis of autism, > 50 with an RC diagnosis of DD (recruitment began later for this group), and > 120 from the general population. By the end of the first 5 years of funding, we expect to have a total of approximately 650–700 children enrolled. Among contacted families of children with autism, 20% were ineligible, 22% refused, and 58% agreed to participate. Among general population families with whom we made contact, 22% were ineligible, 41% refused, and 36% agreed to join the study.

Among children with a diagnosis of autism recruited from RCs, after assessment by CHARGE study personnel, 64% met criteria on both the ADOS and ADI-R. Among those 3 or 4 years of age who are California DDS eligible based on their diagnosis of autism, 64% meet criteria on both instruments, another 9% meet criteria on the ADOS alone, and a further 14% on the ADI-R alone, for a total of 87%. Additionally, among the remainder, 6% meet criteria for ASD based on both examinations (scores at least 7 on ADOS module 1 or at least 8 on ADOS module 2, and meets cutoff in ADI-R for section D and either section A or B, and falls within 2 points on the other section of A or B); another 5% meet criteria for ASD based on either ADI-R alone or ADOS alone. Fewer than 2% would not be classified as being on the spectrum.

Among those recruited through RCs with a diagnosis of DD, the percentage that showed delay in both adaptive and cognitive domains was 64%, with another 6% that met the cutoff on at least one of the tests. Among those who entered the study with a diagnosis of DD, 3% met criteria for autism and another 8% met criteria for ASD.

Another phenotypic distinction we are investigating is early-onset versus regressive autism, as defined by the language and social regression questions on the ADI-R and the CDQ. Using a broad definition of regression that includes loss of previously attained language and/or social skills (Ozonoff et al. 2005), close to 50% of the CHARGE children with a confirmed diagnosis of autism had regression.

Finally, Table 3 provides basic demographic information about the CHARGE study sample, based on data from the birth certificate. This table also provides comparison information about the pool of births from which we recruit the general population controls. Compared with this pool, mothers who participate are older, more highly educated, and more likely to have private health insurance. Participant mothers of general population controls are also more likely to have been born in the United States. The children were more likely to be twins. In further work, the autistic and DD participants will be compared with their respective pools.

### Community partnership

A community advisory council (CAC) was formed early in the development of this project to maximize participation in the research by parents, clinicians, service providers, advocacy organizations, and RC and DDS staff. Parental suggestions regarding the collection of specimens and information from younger siblings of affected children were incorporated into the study design. The CAC meets regularly to hear updates on study progress and provide input. CAC members have given critical advice on data collection instruments, ways to make the clinical protocol as child-friendly and special-needs–friendly as possible, and strategies to enhance recruitment.

## Discussion

The CHARGE study is building an infrastructure that will support multiple investigations of autism and related neurodevelopmental disorders. The psychometric evaluations and clinical examinations combined with extensive exposure information and biologic specimens represent rich resources for research on etiology and phenotypic expression of these disorders and make possible the comprehensive approach needed to advance understanding of autism and DD. In our clinical assessments of > 300 children identified with autism in the California DDS system, we have confirmed the diagnosis in 87%, suggesting that the large increases in DDS system clients with autism over the last decade or two is unlikely to be due to overdiagnosis in younger cohorts.

Although several large birth cohort studies recently initiated or in progress will be able to examine factors that predict autism, the number of cases of autism in the CHARGE study may be comparable with what is expected in birth cohorts of 100,000 (i.e., we have enrolled > 360 children with autism and are continuing recruitment). In contrast with large cohort studies with dispersed populations, we are able to confirm diagnoses using standardized instruments administered by a small, well-trained clinical assessment team. Additionally, in cohort studies attempting to address a wide range of health and developmental outcomes, the exposures and factors measured will not necessarily have been chosen for relevance to autism.

The specimen bank is currently being used by several laboratories that are part of the UC Davis CCEH. In this first stage, xenobiotic and biochemical profiles of children with autism are being compared with those of unaffected children, and comparisons are being made between different autism phenotypes. As distinguishing features emerge, the second stage will be to determine whether any differences in biomarkers were present at birth, using the neonatal blood spots where possible. Data and specimens will be made available to qualified researchers with targeted, worthwhile hypotheses not being addressed by CCEH and CHARGE investigators.

Limitations of this study must be recognized. Much of the information will be gathered retrospectively. The only biologic specimens prospectively collected (i.e., before diagnosis) are the newborn blood spots and, for some children, baby hair locks. Similarly, questionnaires on use of pesticides and other household products will be retrospective and hence subject to reporting/recall bias. Thus, the large birth cohort studies under way or in preparation will complement the CHARGE study by providing fully prospective data, although they are subject to the limitations described above. Nevertheless, in the CHARGE study, medical records will yield prospectively recorded data on treatments, illnesses, and prescription medications. Other unbiased, relevant sources of information on xenobiotics include blood measurements that represent cumulative exposures for persistent compounds and California’s Pesticide Use Reporting system, which documents commercial pesticide applications that can be linked to participant residences during critical time windows.

## Conclusion

Although sporadic studies have identified specific environmental factors that have been associated with autism, no previous effort has attempted to address the broad spectrum of environmental factors that may, in combination with genetic susceptibility, affect development and severity of this condition in the population. The CHARGE study is charting new territory in the investigation of etiologic factors for autism and DD. The goal of the CHARGE study is to understand causes of autism and DD, both genetic and environmental, in order to reduce their incidence in the future. The design combines a large population-based sample of children with different patterns of development; standardized diagnostic assessments of autism, cognitive development, and adaptive behavior by trained assessors; medical and neurologic examinations; detailed reviews of medical records; and an extensive set of questionnaires describing phenotypic characteristics and environmental exposures from preconception through early childhood. Currently, it is unique in its emphasis on environmental factors and its tight linkage with state-of-the-art laboratories of the UC Davis CCEH that enable us to address biologic markers of xenobiotic exposures, immunologic responses, and gene expression. Other features include active community involvement, an ethnically diverse pool of participants, and inclusion of developmentally delayed children in addition to general population controls. Finally, the collaboration by CHARGE study investigators with other population-based autism epidemiologic efforts currently under way, such as the national Centers for Autism and Developmental Disabilities Research and Epidemiology (CADDRE) study, will create valuable opportunities for replication and perhaps data pooling.

## Figures and Tables

**Table 1:** Biospecimen use for susceptibility and exposure markers.^a^

	Child’s blood	Child’s urine	Newborn blood spot	Hair
Immune markers				
Cytokines	X		X	
Immunoglobulins (general)	X		X	
Antigen-specific Ig responses	X		X	
Cell activation	X			
Lipid profiles	X			
Brominated flame retardants	X			
Pesticide metabolites		X		
Metals	X		X	X
Genomics	X			
Genetics	X			

a Not an exhaustive list of assays.

**Table 2:** Data collection protocol for CHARGE study: three developmental groups of children.

Instruments administered	Administered to AU, DD, and GP children (except where noted)
In clinic	
ADOS (Lord et al. 2000)	AU only
ADI-R (Le Couteur et al. 2003)	AU only
MSEL (Mullen 1995)	
VABS (Sparrow et al. 1984)	
SCQ (Rutter et al. 2003)	DD or GP only
Child’s medical history	
Family autoimmune history	
Family medical history	
Physical, neurological, and dysmorphology exams	
CDQ	
Family early developmental characteristics	
Self-administered questionnaires completed at home	
Aberrant Behavior Checklist (Aman and Singh 1994)	
Multiple language questionnaire	
Gastrointestinal disorders survey	
Sleep history survey	
Telephone-administered exposure questionnaire	

Abbreviations: AU, autism; GP, general population.

**Table 3:** Demographics in CHARGE study (%).

	CHARGE study participants			
	AU (*n* = 341)	DD (*n* = 54)	GP^a^ (*n* = 101)	GP pool (*n* = 1,240)
Nonsingletons	6.2	0	3.0	1.6
Mother’s age ≥ 35 years at delivery	25.5	18.5	28.7	16.0
Mother’s education < 12 years	6.8	14.8	12.1	29.8
Mother’s education ≥ 16 years	41.8	27.8	41.4	23.1
Mother born in United States	72.4	68.5	70.3	54.5
Mother born in Mexico	10.3	25.9	14.9	24.1
Mother born outside
United States and Mexico	17.3	5.6	14.9	21.4
Payment method for delivery
Public	17.6	37.0	19.8	42.9
Private	82.4	63.0	80.2	57.1
Male child^b^	88.0	66.7	83.2	79.4

Abbreviations: AU, autism; GP, general population.

a From birth certificates; pool consists of a stratified random sample selected to have 80% boys, to match the overall age distribution of the autism cases, and from the same geographic catchment area as the other two groups.

b The general population pool was selected with odds of 4:1 male-to-female ratio.
